# Hereditary cancer panel testing challenges and solutions for the latinx community: costs, access, and variants

**DOI:** 10.1007/s12687-021-00563-y

**Published:** 2021-11-06

**Authors:** Michael P. Douglas, Grace A. Lin, Julia R. Trosman, Kathryn A. Phillips

**Affiliations:** 1 UCSF Center for Translational and Policy Research On Personalized Medicine (TRANSPERS), Department of Clinical Pharmacy, University of California San Francisco, 490 Illinois Street, 3rd Floor, Box 0613, San Francisco, CA 94143 USA; 2 Department of Medicine, University of California, San Francisco, CA USA; 3 Philip R. Lee Institute for Health Policy Studies, University of California, San Francisco, CA USA; 4 Center for Business Models in Healthcare, Glencoe, IL USA; 5 UCSF Helen Diller Family Comprehensive Cancer Center, University of California-San Francisco, San Francisco, CA USA

**Keywords:** Hereditary cancer panels, Latinx, Access, Cascade testing, Cost

## Abstract

Hereditary breast and ovarian cancers (HBOCs) are common among the Latinx population, and risk testing is recommended using multi-gene hereditary cancer panels (HCPs). However, little is known about how payer reimbursement and out-of-pocket expenses impact provider ordering of HCP in the Latinx population. Our objective is to describe key challenges and possible solutions for HCP testing in the Latinx population. As part of a larger study, we conducted semi-structured interviews with key provider informants (genetic counselors, oncologist, nurse practitioner) from safety-net institutions in the San Francisco Bay Area. We used a deductive thematic analysis approach to summarize themes around challenges and possible solutions to facilitating HCP testing in Latinx patients. We found few financial barriers for HCP testing for the Latinx population due to laboratory patient assistance programs that cover testing at low or no cost to patients. However, we found potential challenges related to the sustainability of low-cost testing and out-of-pocket expenses for patients, access to cascade testing for family members, and pathogenic variants specific to Latinx. Providers questioned whether current laboratory payment programs that decrease barriers to testing are sustainable and suggested solutions for accessing cascade testing and ensuring variants specific to the Latinx population were included in testing. The use of laboratories with payment assistance programs reduces barriers to HCP testing among the US population; however, other barriers are present that may impact testing use in the Latinx population and must be addressed to ensure equitable access to HCP testing for this population.

## Introduction

Next-generation sequencing allows for testing multiple genes in a single test. We address hereditary cancer panel (HCP) testing for hereditary breast and ovarian cancer (HBOC) in the Latinx population. Hereditary cancer testing is now recommended to be conducted using multi-gene HCPs (NCCN 2021). Prior studies show that insurance coverage is variable and increasing for HCP (Trosman et al. 2017), and there have been barriers to access reported for the Latinx population (Hurtado-de-Mendoza et al. 2018; Cruz-Correa et al. 2017). For example, Medicaid expansion has increased coverage for genetic testing, but the impact this policy has had on access to testing in minority populations has not been studied. Furthermore, little is known about how payer reimbursement and out-of-pocket (OOP) expenses impact provider ordering of HCP in the current clinical and reimbursement landscape, particularly related to the Latinx population.

BRCA1/2 pathogenic variants play an important and relevant role in HBOC in the Latinx population (Alvarado et al. 2020; Abul-Husn et al. 2019; Ricker et al. 2016). Thus, HCP testing may be particularly important for this population (Weitzel et al. 2013). Furthermore, research suggests that healthcare providers often lack an understanding that Latino families may be at elevated risk for HBOC (Hurtado-de-Mendoza et al. 2018). Latinx patients are less likely to receive genetic counseling education, referrals, and testing services and have less awareness of genetic testing than non-Hispanic Whites and other minority populations (Cruz-Correa et al. 2017). Despite low awareness, Latinx patients have a high interest in participating in genetic counseling and testing (Hurtado-de-Mendoza et al. 2018; Mai et al. 2014; Hurtado-de-Mendoza et al. 2020).

This study explored providers’ perspectives on key challenges and possible solutions for HCP testing in the Latinx population, using data from a larger interview study (Lin et al. 2021). Specifically, we explored providers’ perspectives on whether Latinx patients had different experiences with test ordering, counseling, and access to testing than other safety-net patients. This study adds to the literature on access to HCP across borders and informs future studies on the need to include ethnicity and location in decision-making processes for patients.

## Materials and methods

### Study population

In this study, we conducted one-on-one semi-structured interviews with five key provider informants—3 genetic counselors, 1 oncologist, and 1 oncology nurse practitioner (henceforth referred to as participants)—from cancer genetics or oncology clinics located within four safety-net clinics in the San Francisco Bay Area. Since California has a large Latinx population, and safety-net clinics provide care to many of these patients (clinics reported that Latinx patients were 30–50% of their population), we focused the study on this population. The institutional review board at the University of California, San Francisco, approved all study procedures.

### Interview guide development

The interviews were part of a larger provider interview study by Lin et al., which focused on assessing the impact of payer reimbursement and OOP expenses on provider ordering of HCP (Lin et al. 2021). This larger study did not examine racial/ethnic differences.

The interview guide included questions about the testing pathway, the impact of payer reimbursement, and OOP expenses on testing decisions, clinician discussion on OOP expenses, challenges and potential solutions to facilitate testing, and the role of direct-to-consumer and hybrid laboratories (that are consumer-facing but offer medical-grade testing ordered through a clinician). As part of these interviews, we asked several questions explicitly addressing testing in the Latinx population with a focus on three topics: (1) Differences in test ordering and counseling, (2) challenges, and (3) potential solutions. In addition to the questions asked in Lin et al. (Lin et al. 2021) , we asked the following:

- What % of their patients are of Latinx descent?
- What is the approximate breakdown of insurance coverage in your clinic (Medicaid, Medicare, private insurance, uninsured)? How does this vary for Latinx patients?
- Do you consider variants of unknown significance when discussing testing or test results with Latinx patients vs. Caucasian patients?
- Do you ever choose a particular test over another when the patient is Latinx? If so, why?
- What impact does insurance coverage have on the process of ordering HCP tests? Specifically, what happens if insurance coverage is denied? What are the other options for offering testing at an affordable cost to the patient? Any specific to the Latinx population?
- Do you see differences in your approach to discussing HCPs with the Latinx population?
- What are challenges related to patient’s benefits, test costs, or copay issues (if any) for ordering HCP testing in your clinic? Are there any challenges specific to Latinx population? What approaches do you use and/or would you recommend to address these challenges?

### Data collection and analysis

Interviews were conducted by two investigators (G. L. and J. T.) and recorded and transcribed verbatim. Transcripts were coded independently by the two investigators using deductive thematic analyses, and themes were identified (Braun and Clarke 2006). Related codes were collapsed into key themes based on the interview guide in an iterative and consensus-based approach. The themes were also stratified based on population (Latinx or non-Latinx). Consensus was reached by discussion among authors.

## Results

We identified three key themes that reflect challenges for HCP testing in the Latinx population: sustainability of low-cost testing and OOP to patients, access to cascade testing for family members, and choosing appropriate tests that include variants found in the population. Participants also suggested solutions to these challenges.

### Sustainability and awareness of low-cost testing, and out-of-pocket test costs, and expenses

There were no differences in OPP test costs for Latinx patients (proband) presenting for testing compared to non-Latinx patients. This was due to the fact that the vast majority of proband patients (both Latinx and non-Latinx) are able to access laboratory payment assistance programs that allow them to obtain the recommended test for little or no OOP test cost. However, participants stated concerns about whether laboratory payment assistance programs were sustainable long-term; if these programs were discontinued, it may impact testing access for the Latinx population, as they are less likely to be insured. Participants stated another barrier to access was patients’ lack of awareness that low and no-cost options were available. Additionally, participants reported that other OPP Expenses such as transportation costs (e.g., gas, public transportation, or parking) to/from clinics were challenging for some Latinx patients.

In response to these challenges, providers suggested better outreach to underserved patients of Latinx descent to raise awareness that testing’s availability and affordability would help increase testing utilization.

*Example Quote: “Transportation can be difficult to my clinic” (Participant 4).*

### Access to cascade testing in family members

Participants stated that a challenge for HCP was access to cascade testing for family members living outside the USA, as these family members face difficulty with both access to testing and cost of testing. Family members living within the US mostly have similar options to the original family member (proband) being tested since companies often offer free cascade testing; however, participants reported some difficulty in access to testing when family members are not located near testing centers.

Participants stated that the most difficult challenge occurs when a pathogenic variant is found, and cascade testing for the family is recommended. They noted the need for education for providers who service the Latinx and other underserved communities to ensure the most appropriate HCP test is ordered. Solutions to both challenges included providing letters or reports in a native language for patients who test “positive” for a variant of significance for family cascade testing, and developing education materials specific for the Latinx population and distributing to clinics serving the population.

Another challenge was patient immigration status. Participants stated that undocumented family members living outside the San Francisco Bay Area in places with limited access to healthcare may not have ready access to testing. One solution suggested for those whose family members are within the USA but not close to genetic services or health care providers or who live outside the USA is the use of laboratories that provide free or low-cost cascade testing by mail, such as Color Genomics. A solution for undocumented family members to access testing regardless was to consult with academic medical centers as some have been successful in obtaining testing for undocumented patients.

*Example Quote: “But there may be patients that are really nervous about that, and may not come in the door...So, I don*’*t really know that because they wouldn*’*t get to me, but San Francisco is really good about covering everyone regardless of their immigration status.” (Participant 1).*

### Choosing appropriate tests for variants found in the population

Participants emphasized the need to use a test that reports variants specific to the Latinx population, especially for the *BRCA1/2* genes. They suggested using HCP tests that analyze the full sequencing of all genes instead of targeted panels that analyze specific pathogenic variants.

*Example Quote: “I*’*ve been ordering the wider panels for everybody. But I have been noticing that there are certain variants within the BRCA genes that are more common for Latina populations versus other populations. So it*’*s important to have full sequencing of all of the genes instead of just looking for specific mutations.” (Participant 4)*

Participants also stated that counseling in the Latinx population is geared toward more unknowns or findings that are not understood due to limited studies in this population, and as a result have more variants of unknown significance.

*Example Quote: “In your particular population we don*’*t know as much about, so we do tend to find things we don*’*t understand. So I guess, that would*’*ve been a little bit of a difference then with counseling.” (Participant 5)*

An additional challenge with post-test counseling Latinx patients is the availability of direct-to-consumer testing such as 23andme, since this kind of test does not detect all possible pathogenic variants for HBOC related genes (only two variants in the *BRCA1* gene and one variant in the *BRCA2* gene that are most commonly found in the Ashkenazi Jewish population). Therefore, Latinx and non-Latinx patients who have had testing done through 23andme, while not a clinical test, may believe they have had adequate testing for HBOC and need additional post-test counseling about the shortcomings of direct-to-consumer tests and the need for further testing.

*Example Quote: “if you have a Latina person who*’*s taking the 23andMe test and their mutations are very different from Ashkenazi mutations, if the test isn*’*t going to catch their VRCA…So I do really warn them, Don*’*t read in to heavily to your results. And if you do, bring them to me and we can talk about them.” (Participant 4)*

## Discussion

We identified three key themes reflecting challenges for HCP testing in the Latinx population compared to other groups: sustainability of low-cost testing and OOP expenses, access to cascade testing in family members, and choosing appropriate tests that include variants found in the Latinx population. Our study has implications for clinical practices of providers serving Latinx patients, as well as future research of adoption of HCPs in this population.

### Sustainability and awareness of low-cost testing, and out-of-pocket test costs, and expenses

Lin et al. (Lin et al. 2021) and Erwin et al. (Erwin et al. 2020) both report equitable access to testing within the USA for Latinx and non-Latinx patients provided they could access the health care system. These studies found equitable access was primarily due to laboratory pricing models that include low or no OOP test costs for patients. This is in contrast to earlier studies (Kinney et al. 2010; Vadaparampil et al. 2010; Vadaparampil et al. 2011) and demonstrates that laboratory business models can lower previously reported barriers to testing. However, the sustainability of these programs is unknown, and there is concern about widening disparities if these programs should be discontinued (Scheuner et al. 2021).

Additionally, there is the need to address specific barriers around other OPP expenses and other challenges for the various Latinx subpopulations in the USA (Ricker et al. 2018). Specifically, a prior study found that Cubans mostly identified financial concerns as their primary barrier to testing, whereas Mexicans reported a lack of provider discussion, and Puerto Ricans reported fear of tests and lack of awareness (Kinney et al. 2010; Vadaparampil et al. 2010; Vadaparampil et al. 2011). Developing patient-centered education materials that address the concerns of specific Latinx subpopulations may help outreach to those populations, as well as materials that address free and no-cost testing programs.

### Access to cascade testing in family members

We found that cascade testing for Latinx family members, particularly those outside the USA, remains a significant barrier. Two main issues arise, accessing a place to get tested and communicating test results, particularly about pathogentic variants for family members who do not speak English so that they can get the appropriate test. In terms of access to testing, medical genetic services in Latin America are provided mainly by the public sector, but the service is uneven and concentrated in affluent and urban areas; for example, it has been reported that it is challenging to get testing in Mexico and other countries (Hurtado-de-Mendoza et al. 2018; Roberts et al. 2018). Additionally, beyond geography, the cost of genetic testing in Latin America makes this service inaccessible to a great majority of the population (Cruz-Correa et al. 2017). For family members who do not speak English, providers have been able to alleviate some access issues by providing their patients a letter or report in the appropriate language about the variant that was found.

Our findings are similar to Hurtado-de-Mendoza et al., who reported that language barriers, family communication, and testing relatives who live outside the USA were important aspects to consider when working with at-risk Latinx populations (Hurtado-de-Mendoza et al. 2018). Furthermore, a review by Roberts (Roberts et al. 2018) reported that major barriers to cascade screening delivery included suboptimal communication between the proband and family and geographic obstacles to obtaining genetic services. Our results confirm that these issues are barriers to testing for the Latinx population. A study by Caswell-Jin evaluated the use of an online initiative for cascade testing of relatives for hereditary cancer risk including HBOC, and found that an online, low-cost program is an effective approach to implementing cascade testing, and that up to 5% of the general population may carry a pathogenic variant in 1 of 30 cancer-associated genes (Caswell-Jin et al. 2019).

### Choosing appropriate tests for variants found in the population

Participants noted that the main challenge to ordering HCP tests in the Latinx population was understanding that different pathogenic variants are found in the Latinx population. Some targeted panel tests may not detect applicable variants found in the Latinx population. For example, there are differences in the location and type of variants in the *BRCA1/2* genes based on Latinx population. Lynce et al. (Lynce et al. 2016) described pathogenic variants found in various Latinx subpopulations (e.g., Mexican, Caribbean, Central, and South American), while Dutil et al. (Dutil et al. 2015) described the spectrum of *BRCA1/2* alleles in Latin America and the Caribbean, and Ossa and Torres (Ossa and Torres 2016) provided a review of the founder and recurrent mutations in *BRCA1/2* genes in Latin American Countries. These studies support the need to educate providers who service the Latinx and other underserved communities to ensure the most appropriate genetic test is ordered.

### Limitations

Our interviews were limited to safety-net clinics serving the Latinx population in the San Francisco Bay Area and in sample size. However, the key findings serve as an impetus for future studies in other regions or nationally and informs the development of a nationwide study addressing access in the Latinx populations. We do acknowledge that California, in general, has much better healthcare access for individuals who are un- or underinsured and/or undocumented than other parts of the USA. Additionally, the majority of our key informants were genetic counselors (3 genetic counselors, 1 oncologist, and 1 oncology nurse practitioner), as they are the providers who mainly see patients referred for Hereditary Cancer Testing; other types of providers may identify additional barriers and solutions. Furthermore, non-genetic providers are more likely to be unaware of some programs discussed (e.g., no- or low-cost testing) and unable to utilize such programs for their patients.

## Conclusions

The use of laboratories with payment assistance programs reduces barriers to hereditary cancer panel testing among populations living in the USA, including the Latinx population. However, other barriers exist and must be addressed to ensure equitable access, such as the sustainability of low-cost testing, access to cascade testing for family members, and understanding of variants found in the Latinx population. The participants suggested several solutions such as the need to educate providers who service the Latinx and other underserved communities to ensure the most appropriate HCP test is ordered, and patient-centered education materials that address the concerns of specific Latinx subpopulations may help outreach to those populations as well as materials that address free and no-cost testing programs in both English and Spanish. Researchers, laboratories, and other stakeholders interested in increasing knowledge about variants in the Latinx population should consider exploring ways to conduct outreach to Latinx populations in the USA and improve testing access outside the USA.

## Data Availability

Data and material are available via reasonable request from corresponding author.

## Declarations

### Disclaimer

The California Initiative to Advance Precision Medicine, the National Human Genome Research Institute, and the National Cancer Institute had no role in the preparation, review, or approval of the manuscript or decision to submit the manuscript for publication.
